# An efficient graph attention framework enhances bladder cancer prediction

**DOI:** 10.1038/s41598-025-93059-5

**Published:** 2025-04-01

**Authors:** Taghreed S. Ibrahim, M. S. Saraya, Ahmed I. Saleh, Asmaa H. Rabie

**Affiliations:** Computers and Control Dept. faculty of engineering, Mansoura University, Mansoura, Egypt

**Keywords:** Bladder cancer, Graph convolutional neural network (GCNN), Cancer prediction, Attention mechanism, Driver genes, Cancer, Computational biology and bioinformatics, Engineering, Materials science, Mathematics and computing

## Abstract

Bladder (BL) cancer is the 10th most common cancer worldwide, ranking 9th in males and 13th in females in the United States, respectively. BL cancer is a quick-growing tumor of all cancer forms. Given a malignant tumor’s high malignancy, rapid metastasis prediction and accurate treatment are critical. The most significant drivers of the intricate genesis of cancer are complex genetics, including deoxyribonucleic acid (DNA) insertions and deletions, abnormal structure, copy number variations (CNVs), and single nucleotide variations (SNVs). The proposed method enhances the identification of driver genes at the individual patient level by employing attention mechanisms to extract features of both coding and non-coding genes and predict BL cancer based on the personalized driver gene (PDG) detection. The embedded vectors are propagated through the three dense blocks for the binary classification of PDGs. The novel constructure of graph neural network (GNN) with attention mechanism, called Multi Stacked-Layered GAT (MSL-GAT) leverages graph attention mechanisms (GAT) to identify and predict critical driver genes associated with BL cancer progression. In order to pick out and extract essential features from both coding and non-coding genes, including long non-coding RNAs (lncRNAs), which are known to be crucial to the advancement of BL cancer. The approach analyzes key genetic changes (such as SNVs, CNVs, and structural abnormalities) that lead to tumorigenesis and metastasis by concentrating on personalized driver genes (PDGs). The discovery of genes crucial for the survival and proliferation of cancer cells is made possible by the model’s precise classification of PDGs. MSL-GAT draws attention to certain lncRNAs and other non-coding elements that control carcinogenic pathways by utilizing the attention mechanism. Tumor development, metastasis, and medication resistance are all facilitated by these lncRNAs, which are frequently overexpressed or dysregulated in BL cancer. In order to reduce the survival of cancer cells, the model’s predictions can direct specific treatment approaches, such as RNA interference (RNAi), to mute or suppress the expression of these important genes. MSL-GAT is followed by three dense blocks that spread the embedded vectors to categorize PDGs, making it possible to determine which genes are more likely to cause BL cancer in a certain patient. The model facilitates the identification of new treatment targets by offering a thorough understanding of the molecular landscape of BL cancer through the integration of multi-omics data, encompassing as genomic, transcriptomic, and epigenomic metadata. We compared the novel approach with classical machine learning methods and other deep learning-based methods on benchmark TCGA-BLCA, and the leave-one-out experimental results showed that MSL-GAT achieved better performance than competitive methods. This approach achieves accuracy with 97.72% and improves specificity and sensitivity. It can potentially aid physicians during early prediction of BL cancer.

## Introduction

Bladder cancer is the 10th most prevalent cancer globally, with nearly 600,000 cases diagnosed annually. Despite advancements in prediction and treatment, the five-year survival rate remains low at 77% due to late diagnosis^1^. Prediction systems aim to improve diagnostic accuracy and avoid unnecessary medical examinations. Machine learning has gained attention in cancer prognosis due to its high efficacy in precision medicine projects^2^. Traditional computational methods face challenges due to the complex nature of genetic data. Deep learning methods like multi-layer perceptron, convolutional neural networks, and autoencoders are often used in cancer prognosis, showing potential in various applications^3,4^. Understanding the genetic mechanisms driving breast cancer (BL) is crucial for personalized treatments and improving outcomes. Advances in genomics have highlighted the roles of both coding and non-coding genes in cancer progression^5,6^. Non-coding genes, such as lncRNAs, regulate gene expression, tumorigenesis, and cancer prediction. Integrating both coding and non-coding genomic information is essential for accurate cancer driver gene identification and prediction. Advances in computer technology and sequencing approaches are focusing on cancer driver gene discovery, using mutation-based techniques and network-based techniques like Driver Net and HotNet2^7,8^.

Bladder cancer is a complex disease characterized by molecular and genetic changes that impact tumor behavior, prognosis, and development. Identifying driver genes that initiate and promote cancer progression is crucial for personalized cancer prediction^9^. Coding genes, which make up 1–2% of the human genome, produce proteins for cellular survival and function. Gene mutations can cause abnormal protein function, unchecked cell division, apoptosis evasion, and genomic instability. Non-coding genes, like microRNAs and long non-coding RNAs, control gene expression and regulate gene regulatory networks^10^. Epigenetic changes in bladder cancer can activate or repress important driver genes, leading to poor prognosis, metastasis, and bladder cancer development. Biomarkers like long non-coding RNAs are becoming available for early cancer detection, prognosis, and treatment response. Combining coding and non-coding genetic data is necessary for personalized driver gene prediction for bladder cancer, focusing on important driver genes using network analysis, graph convolutional networks, and attention processes. This approach can lead to targeted therapies and advanced medical strategies for bladder cancer^11^.

The study proposes a novel approach for BL cancer prediction using graph attention networks (GATs), a variant of GNNs. MSL-GAT introduces multiple stacked layers of GAT with regularization techniques to improve model performance. The approach establishes a patient-specific gene interaction network, focusing on relevant genes and relationships. GAT assigns attention weights to each gene’s neighbors, prioritizing influential interactions. This multi-scale, relation-aware framework captures complex dependencies between genes and their interactions, leading to more accurate predictions of cancer driver genes and patient-specific outcomes in BL cancer. The approach is complemented by three dense blocks, providing more flexibility to pick up complex interactions and relationships in embeddings. This paper aims to demonstrate the effectiveness of integrating coding and non-coding genes into a GNN-based framework with GAT. The remainder of this work is structured as follows: Sect. 2 displays earlier research on the significance of BL tumor prognosis and genomic tests. The proposed GCNN with an attention mechanism to detect PDG and predict BL cancer is the focus of Sect. 3. The experimental finding is shown in Sect. 4. The paper’s conclusion and future plans are outlined in Sect. 5.

## Related work

This section will cover prior studies on BL cancer prognosis techniques. BL cancer, a prevalent and costly disease, is causing a global increase in new cases. Early detection improves 5-year survival rates by 80% or more. Deep learning techniques have emerged to handle complex data, such as genomic data, in BL cancer prognosis. Genomic data can predict patient outcomes and tailor treatments to individual patients, making it a valuable tool for understanding the disease’s etiology and prevention strategies. In^12^, the Bladder Cancer Prediction Using Genetic Algorithm and Fuzzy Rule-Based System (BLCP-GAFR) model combines gene expression and methylation data to predict bladder cancer. It employs genetic algorithms to identify the most effective feature collection and uses a fuzzy rule-based system for 100% accuracy. The model identifies biomarker genes for cancer identification, ensuring the most significant features are selected. However, it faces overfitting risk, complexity of GA, dependence on differential expression and methylation analysis, and lack of validation on independent datasets. In^13^, Bladder cancer gene expression prediction with explainable algorithms (BLCP-XA) uses data from tumoral and non-tumoral tissues to categorize individuals with bladder cancer and identify genes that function well in both tumoral and normal tissues. The study uses interpretable techniques like permutation feature importance (PFI), anchor approaches, Shapley additive explanation (SHAP), and local interpretable model-agnostic explanations (LIME) to promote transparency in gene categorization. XA offers an alternative approach to gene influence and provides a thorough examination of gene relevance, helping physicians make decisions about therapy and prognosis based on clinical evidence. However, XA has limitations, such as its application only to microarray data, potential overfitting, inability to integrate other data types, and lack of external validation.

In^14^, the study (GNN-Surv) uses Graph Neural Networks (GNNs) to predict survival in cancer patients using clinical and genomic data. The models, which combine Probability Mass Function (PMF) and logistic hazard survival approaches, outperform conventional multilayer perceptron models in two urologic cancer datasets. They achieve performance gains of up to 14.6% and 7.9% in the time-dependent concordance index under different graph-building hyperparameters. The study also incorporates patient similarity networks to identify complex interactions between patients using genomic and medical data. However, this method is limited to certain types of tumors and sensitive to hyperparameters. In^15^, The Synthetic Lethality Graph Neural Network (SLGNN) is a model that predicts interactions between synthetic lethal genes (SL) based on cancer types. It divides the SL database into eight subgroups for individual prediction, classifying each SL gene pair based on potential human malignancies. The model uses weighted summation and GNN-based message aggregation to minimize distance correlation and ensure independence in analysis. SLGNN has advantages such as identifying associations among genes, improving prediction quality, and compensating for cancer heterogeneity. However, it faces interoperability issues, limited generalizability across cancer types, difficulty of knowledge graphs, overfitting, and scalability problems. In^16^, Deep Network Learning Cancer (DNLC) is a method used to classify complex diseases like leukemia, colon, lung adenocarcinoma, and squamous cell carcinoma. The Deep Neural Learning Cancer Prediction Model (DNLC) uses five cancer datasets to predict survival rates. Despite its effectiveness, concerns remain about its accuracy. The DNLC model uses relevant features from datasets, trains a deep neural network using genetic or clinical data, and evaluates its early cancer detection capabilities. With an average accuracy of 93%, DNLC is flexible, adaptive, and efficient in processing large datasets. However, it faces challenges such as interpretability, overfitting, computing power requirements, data imbalance issues, and data quality.

In^17^, genome deep learning (GDL) is a novel approach for cancer diagnosis based on genomic variation, using a deep neural network in the TensorFlow framework. Researchers developed 12 specific, total-specific, and mixture-specific cancer identification models, which are better than conventional cytological identification as they detect cancer early and are unaffected by diagnostic tools. GDL can be applied alongside non-invasive prenatal diagnostics and liquid biopsies. However, the models cannot accommodate more cancer types or incorporate additional elements beyond genetic differences. Future developments in biology, deep learning, mass high-reliability variations, and algorithms will address these issues. In^18^, in order to create predictive models, this study used clinical laboratory data with machine learning techniques on 1336 patients with prostate, renal, uterine, BL, and cystitis cancer. Decision trees, random forests, support vector machines, extreme gradient boosting, and mild gradient boosting machines (GBM) were the five machine learning models that were used. Eight clinical laboratory tests were chosen using a two-step feature selection process, and we developed a light GBM BL cancer prediction model. Additionally, this study employed sampling approaches to rectify the unbalanced data. Our research showed promise for cancer detection using clinical laboratory data. In^19^, the study developed a diagnostic signature and an endothelial cell prognosis index (ECPI) by subtyping bladder cancer patients according to gene expression patterns associated with endothelial cells (EC). These technologies help with medication sensitivity assessment, diagnosis, and prognosis prediction. Immunomodulating effects and a worse prognosis were associated with elevated EC levels. Three hub genes with the strongest affinities for curcumin and doxorubicin were found by the ECPI, indicating a direct connection between EC and BC. In^20^, in order to predict lymph node metastasis (LNM) in patients with bladder urothelial carcinoma (BUC) undergoing radical cystectomy, the study set out to create and evaluate machine learning models. Patient data was gathered and split into training and testing sets at random. Prediction models were developed using five machine learning methods, and accuracy and AUC were utilized to evaluate performance.

In^21^, a machine learning study has developed a diagnosis model for bladder cancer (BC), ranking ninth globally. The model uses mitochondria-related genes (MRGs) to identify essential genes for diagnosis, including TRAF3IP3, NMT1, OXSM, and GLRX2. The model’s diagnostic value was validated in datasets GSE13507, GSE3167, and GSE37816. The study also validated the expression of NMT1 in BC cells and its potential clinical use. Further research is needed to confirm its efficacy. In^22^, a study developed a multiple programmed cell death index (MPCDI) for bladder cancer patients based on 1911 PCD-related genes and 19 PCD patterns. The prognosis was better for low MPCDI patients and worse for high MPCDI ones. The nomogram was more accurate than other factors, demonstrating the heterogeneity of BLCA patients. MPCDI scores can guide chemotherapy, with higher scores demonstrating greater effectiveness. In^23^, a novel system combines molecular biological techniques to detect gene mutations in gigapixel Whole Slide Images (WSI). The system uses supervised learning, contrastive learning, and hierarchical deep multi-instance learning to classify mutations, cluster malignant patches, and segment cancerous areas, outperforming existing techniques in experimental data. In^24^, one important therapeutic target for bladder cancer is the fibroblast growth factor receptor (FGFR) pathway; patients with mutations in this system respond well to FGFR-targeted treatments. Research used a logistic regression model and computationally extracted imaging biomarkers from tumor diagnostic slides in order to predict FGFR changes in bladder cancer. In^25^, this study uses systems biology to study advanced bladder cancer and muscle-invasive bladder cancer, identifying potential therapeutic targets for systems drug development. Researchers built candidate GWGENs and designed chemical medication combinations using a deep neural network-based drug-target interaction model. The study offers promising treatment alternatives for both types of cancer. Additionally, the study predicts bladder tumor stages using RNA-sequencing analysis and high-definition CT imaging, discovering a four-gene signature and three-factor radiomics signature.

## The proposed an efficient graph attention framework for bladder cancer prediction (BLCP-GAT)

**Fig. 1:**
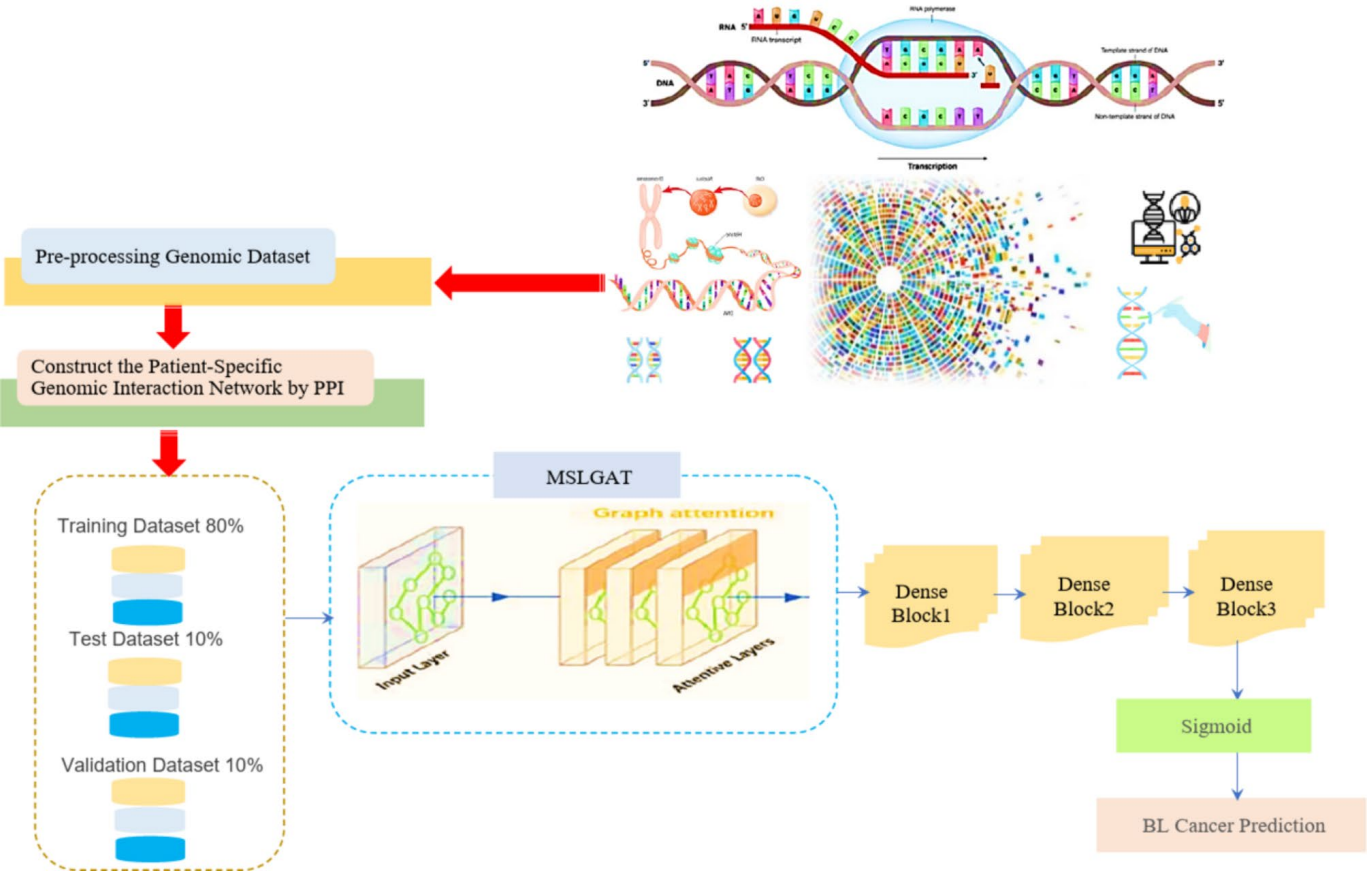
The proposed efficient graph attention framework for bladder cancer prediction.

This section presents further details on the proposed approach of GNN that enhances the identification of driver genes at the individual patient level by leveraging multi-scale attention GAT. A novel approach of GAT will be updated embeddings for both coding and non-coding genes that capture the most critical features in the network. Multi-stacked layered (MSL-GAT) will be enhanced graph-level embeddings that provide insights into the relationships between coding and non-coding genes in the network. The proposed model can capture more complex interactions and dependencies in the genomic data. This is particularly important for BL cancer, where non-coding elements may play critical roles in cancer prediction and progression. The inclusion of non-coding genes ensures that the model captures all relevant genetic information, potentially leading to more accurate identification of driver genes and more personalized prediction and treatment strategies. This novel approach should improve the model’s sensitivity to important nodes (genes) and relationships (edges) within the patient-specific network. This technique, followed by three dense blocks that get the final prediction of BL cancer depending on the presence or absence of driver genes, should improve the accuracy and robustness of personalized driver gene identification in bladder cancer using genomic data from TCGA, as shown in Fig. 1.

### Preprocessing genomic dataset

Preprocessing data is crucial and the first step, as it guarantees that the genomic data is wisely captured and structured so GCN may learn from it efficiently. Through incorporating elements of both coding and non-coding genes, the processing of different kinds of genomic data (such as mutations, expression, CNVs, and methylation), and the creation of a network tailored to one patient in particular, the model is able to gather detailed and individualized information on individual patient genomics. Data preprocessing consists of procedures like: (i) Data, which includes both coding and non-coding genes in this analysis^26^. The raw datasets include information on all relevant genomic elements, such as non-coding RNAs (e.g., microRNAs, long non-coding RNAs), regulatory regions, etc. (ii) Process Genomic Data, including: (a) Somatic Mutation Data (MAF files), from which mutation data is extracted for both coding and non-coding regions. The mutation status is encoded for each genomic element as binary values (“1” for mutation present and “0” for no mutation). Only non-synonymous mutations (e.g., missense, nonsense, and frameshift mutations) were included, as they are more likely to impact protein function. A minimum mutation frequency of 5% across samples was required to include a gene in the analysis, ensuring relevance to the cohort. Both coding and non-coding mutations were considered. (b) RNA-Seq data is processed to obtain expression levels for both coding and non-coding RNAs. Normalization of RNA-Seq data using the log2 transformation and scaling using Min Max Scaler. Identify differentially expressed genes (DEGs) and differentially expressed non-coding RNAs (DENs) specific to the disease. Differentially expressed genes (DEGs) and non-coding RNAs (DENs): To identify differentially expressed genes (DEGs) and differentially expressed non-coding RNAs (DENs), RNA-Seq data is used from the TCGA-BLCA dataset. The following criteria were applied: Threshold for Differential Expression: Genes and non-coding RNAs with an absolute fold change and a false discovery rate (FDR) *< 0.05*(adjusted using the Benjamini-Hochberg procedure) were considered significantly differentially expressed. Statistical Method: Differential expression was calculated using DESeq2, a widely accepted tool for RNA-Seq analysis, to ensure robust identification of DEGs and DENs. (c) Copy Number Variation (CNV) Data: CNV data for both coding and non-coding regions is processed using a method like Genomic Identification of Significant Targets in Cancer (GISTIC2) to identify significant regions of the genome that are subject to copy number. (d) DNA Methylation Data, including methylation data for both coding and non-coding regions. Methylation levels are represented as beta values, which are continuous variables between 0 and 1^27^. (iii) Integration of Coding and Non-Coding Genomic Elements, which involved the following steps: (a) Union of DEGs and DENs: Coding and non-coding elements meeting the differential expression thresholds were combined. (b) Addition of Mutated Genes: Mutated genes passing the above criteria were added to create a comprehensive subset of relevant genomic elements. (c) Statistical Prioritization: Elements appearing in both differential expression and mutation data were given higher weight in downstream analysis, as these are more likely to represent driver events. (iv) Pathway and Functional Enrichment: To validate the biological relevance of the selected elements, we performed pathway enrichment analysis using tools such as Wiki Pathways and Reactome. This confirmed that the identified elements were enriched in pathways critical to bladder cancer, such as PI3K-Akt signaling and p53 signaling.

### Coding gene and non-coding element selection and network construction

This stage is vital for adjusting the analysis to the individual patient, clarifying the data, and establishing a base for precise and customized cancer driver gene predictions. It is including, (i) Identify Relevant Genomic Elements: (a) Mutated Genomic Elements, which mutated coding and non-coding regions are identified for each patient using the processed mutation data. (b) DEGs and DENs for the disease and specific to each patient, which mutated and differentially expressed elements are combined and merged with the list of mutated coding/non-coding regions with DEGs/DENs to create a subset of relevant genomic elements for each patient^28^. (ii) Construct the patient-specific genomic interaction network by protein-protein interaction (PPI) network integration. (a) The PPI network is extended to include interactions between non-coding RNAs and proteins (if available) or uses a more comprehensive network that includes interactions between different types of genomic elements. (b) Build the network, which contains nodes that can represent coding genes, non-coding RNAs, and other regulatory elements. Edges represent interactions between these genomic elements. (c) Create node features, including:

- Molecular Features: Extract features from somatic mutation, methylation, CNV, and RNA-Seq data for each genomic element (coding and non-coding).
- System-Level Features: Incorporate system-level features relevant to both coding and non-coding elements. These could include gene length, number of protein domains (for coding genes), RNA stability, etc.
- Network Structure Features, by applying Node2vec, which is an algorithm designed to learn continuous feature representations (embeddings) of nodes in a graph for the extended network (coding and non-coding elements).

A patient-specific gene interaction network where nodes represent genes and edges represent interactions between these genes based on the PPI network.

#### Construct feature matrix (X) and adjacency matrix (A)

Feature Matrix ($\:X$) is formed by combining molecular, system-level, and network structure features for each genomic element (coding and non-coding). Normalize the features $\:X$ to ensure consistency in scale (values between 0 and 1). Each row corresponds to a specific genomic element (a coding gene, a non-coding RNA, etc.). If there are $\:N$ genomic elements in your network, your feature matrix $\:X$ will have n rows. Each column corresponds to a specific feature associated with the genomic elements. The dimensions of the matrix $\:X$ are typically $\:\left(N*F\right)$, where $\:N$ is the number of nodes in the graph, and $\:F$ is the number of features per node. Based on the patient-specific genomic interaction network, construct the adjacency matrix $\:A$,which represents the graph structure and is an ($\:N*N$) matrix, where ($\:N$) is the number of nodes. The element $\:{A}_{ij}$ represents the interaction between genomic element $\:i$ and genomic element $\:j\:$. It also indicates the presence and sometimes the weight of an edge between node ($\:i$) and node ($\:j$). This matrix defines how information is propagated between nodes in the graph. The feature matrix $\:X$ and adjacency matrix $\:A\:$for each patient will serve as the inputs to the GAT^29,30^.

### Multi stacked-layered graph attention network (MSLGAT)

**Fig. 2:**
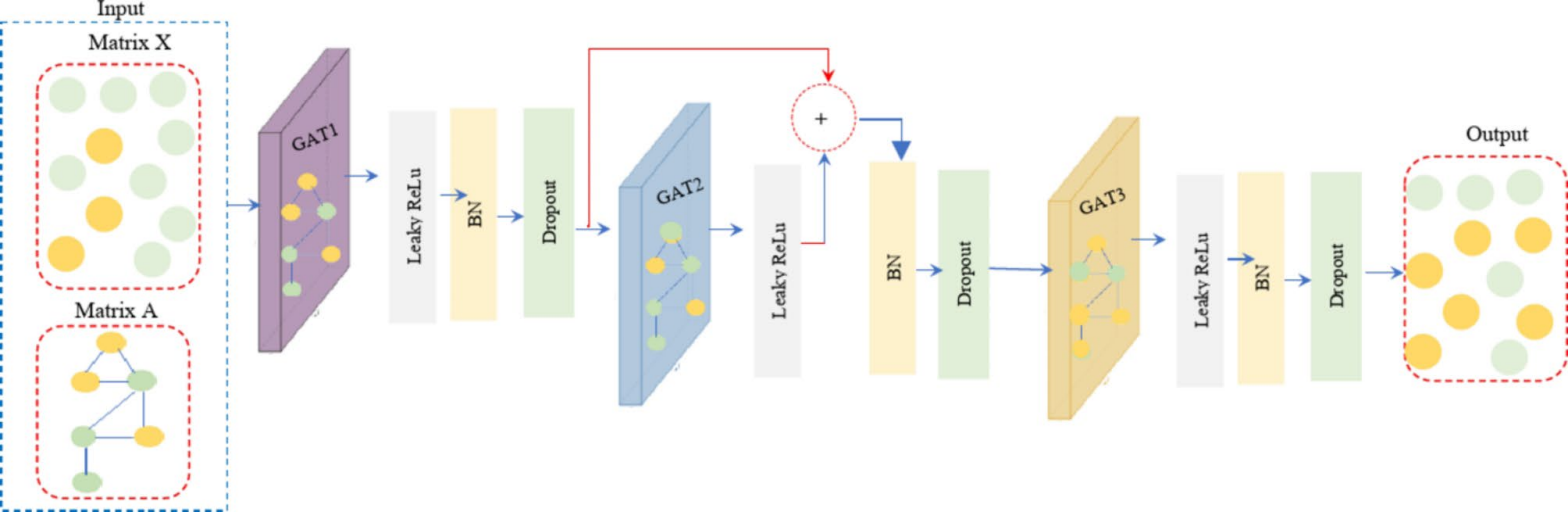
The proposed multi- stacked layers GAT(MSL-GAT).

In this novel approach (MSLGAT), a three-layered Graph Attention Network (GAT) is constructed to extract feature embeddings from both coding and non-coding genes. The objective is to use attention mechanisms to leverage gene-gene correlations in order to identify PDGs. The framework architecture is broken down layer by layer, where each layer improves the representations by taking into account the connections between node characteristics (edges in the graph) and node features (genes). In the MSLGAT method, we proposed three GAT layers, as shown in Fig. 2. The number of GAT’s layers depends on various maxims, including the complexity of the dataset in the task and the interactions that will be predicted. The first reason is capturing multi-hop neighborhood information, which each GAT layer aggregates from neighboring nodes. By applying deeper layers, distant connections and relationships can be captured. One layer can only capture immediate neighbors, two layers can capture neighbors of neighbors, and three layers can capture further interactions. In this task of predicting BL cancer based on detecting the PDGs of each patient, there is a heterogeneous gene (coding and non-coding genes); it is considered the second reason for the decision of the three GAT layers. Utilization of three layers can effectively capture complex relationships and heterogeneous interactions between coding and non-coding genes. For instance, relationships between non-coding RNA and a coding gene might be mediated by other factors (proteins, regulators), which can be captured with more GAT layers. The third reason is needing to achieve the balance between model depth and its performance. when employing more layers may result in the issue of over-smoothing, where node embeddings start to look too similar and lose the diversity of information. Over-smoothing is a widely recognized issue related to graph neural network (GNN) learning, where embedding properties learned by GNNs rapidly grow similar/indistinguishable as layers increase, rendering them unable to distinguish between various networks. GAT layers achieve a compromise between capturing intricate relationships and handling over-smoothing or information loss. Applying one or two layers of GAT doesn’t fully capture the multi-hop relationships existing in genes. In BL cancer genomic data, the implications of gene mutations often propagate across multi-layers of biological regulation, which needs more than two layers to fully capture. The fourth reason is graph depth and information propagation. The type of graph plays a vital role in the number of layers. If the graph is considered a shallow graph, which consists of shallow interactions and connections between genes, GAT with one or two layers is applied. For example, a small, local neighborhood might be captured in just one layer. In contrast, a complex graph with complicated relationships, as in this task, needs three GAT layers. Depending on the nature of BL cancer genomic data, it is effective to implement three layers. The last one is a size of graph (number of nodes and edges). Smaller graphs, which have few nodes and edges, are using fewer GAT layers. It can be effective, as most information is already covered in a small number of hops. On the other side, large graphs with many nodes and edges (containing many gene interactions) need three or four layers. It may be necessary to adequately capture distant node interactions and provide enough depth to propagate information across the graph.

In MSLGAT, there are GAT1, GAT2, and GAT3.Each layer of them has eight multi-head attentions. The multi-head attention technique enables the GAT to concentrate on various features of the neighborhood of a node by utilizing multiple independent attention strategies in parallel. Each head calculates a separate attention score, and the outcomes are either concatenated or averaged to get the last output. The GAT model uses multiple attention heads to enhance its expressiveness and capture intricacies between nodes. Each head can focus on several aspects and areas of the neighborhood, enabling the model to capture a broader set of interactions and dependencies. This reduces learning variance by functioning as a separate estimate of the significance of nearby nodes. Additionally, the model is more adaptable to biological data, such as that seen in the BL cancer prediction challenge involving both coding and non-coding genes, since it acquires a variety of representations. The flexibility of the model is increased by the fact that multiple heads can pick up different interaction patterns. Other heads continue to identify significant connections, which strengthens the model overall and makes it less susceptible to individual failures or data noise. Taken as a whole, the GAT model improves the model’s resilience, expressiveness, and learning variance.

In general, more attention heads are typically applied in shallow layers, as in GAT1, because the model is still learning to pick up relevant features from the raw input. More heads assist in discovering a wide range of connections, relationships, and patterns. On the other side, in deeper layers, as in GAT2 and GAT3, the model has already learned some key patterns, so fewer or the same number of heads may be used. The model concentrates on boosting the learned attributes and capturing higher-level relationships. The number of heads-attention is chosen based on many factors: (i) balancing model expressiveness and complexity so that the model with more heads captures neighborhood interactions better but requires more memory and processing power. In this work, eight heads balance expressiveness and efficiency, allowing for efficient representation without high computing costs. (ii) capturing diverse relationships, which eight heads can focus on different aspects of gene relationships in complex biological networks like PDG prediction in BL cancer. This concept is particularly beneficial in genes, where the diversity of relationships is vital for accurate forecasts, especially in regulatory or protein-protein interactions. (iii) node and feature complexity, as the complexity of the input data, especially when capturing multi-scale interactions between coding and non-coding genes, determines how many heads are included in the genomic data. Graphs with a lot of features or varying neighbors require more heads. iv) avoiding overfitting, which underfitting of the model might result from concentrating on a small number of relationships or missing significant interactions. In contrast, overfitting occurs when the dataset is insufficient to support the addition of more parameters. With eight attention heads, a wide range of interactions are captured without excessively complicated parameterization, offering a balanced capacity to generalize. (v) computational resources, which eight attention heads ensure that a wide range of node interactions are captured while maintaining the computational viability of the model despite a rise in computational workload. (vi) impact on dimensionality and information flow that the dimensionality of the output is influenced by the number of heads in stacked layers, which improves the model’s ability to convey intricate patterns. As demonstrated in the first GAT layer, averaging outputs from many heads in deeper levels guarantees smoother information flow across layers and decreases complexity. (vii) the nature of the task that will be processed, which eight heads are essential for recording intricate gene interactions across several linkages in order to detect PDG in BL cancer. For deeper features or complex interactions, use more heads; for simpler relationships, use fewer heads to guarantee that all relevant data is captured.

In our proposal, in the first layer (GAT1), different patterns are learned from node features and their closest neighbors using eight attention heads. This assists in capturing a wide range of connections and relationships at the early stages or initial level. In the second layer (GAT2), the model integrates data from two-hop neighbors to expand on the properties identified in the first layer. This is essential, as a lot of gene interaction levels must be taken into account when analyzing genomic data. In the third layer (GAT3), by merging data from nodes three hops away, the model boosts embeddings and makes sure it accurately depicts the variety of relationships as it delves deeper into graph architecture.

#### GAT layer 1

In the first layer of MSL-GAT, the input is depicted as: (i) Node Features (matrix $\:X$), which include details about the expression profile of each gene and other genomic features (such as somatic mutations, expression data, copy number variations, or methylation data). Each feature vector’s dimensionality is related to the total number of features associated with every gene. (ii) Adjacency Matrix (matrix $\:A$), which presents the network of gene relationships, where every entry denotes whether a relationship, or edge, exists between two genes or not. GAT1 with eight head attentions detects which genes that are nearest in proximity are most crucial for updating the representation of each node. In order to accomplish this, it first calculates the attention coefficient ($\:{\alpha\:}_{ij}$) for each nearby node before applying the learned weights to their features. The model is able to capture various elements of the neighborhood’s effect because the multi-head attention mechanism duplicates this process over eight attention heads. The attention coefficients $\:{(\alpha\:}_{ij}$) ​ between nodes $\:i$ and $\:j$ are computed as in (1)^30^:

1 $$\alpha _{{ij}} = \frac{{\exp \left( {Leaky\text{Relu} \left( {\acute{a} ^{T} \left[ {W\;h_{i} \parallel W\:h_{j} } \right]} \right)} \right)}}{{\sum\nolimits_{{k \in N\left( i \right)}} {\exp \left( {Leaky\text{Relu} \left( {\acute{a} ^{T} \left[ {\left[ {W\:h_{i} \parallel W\:h_{k} } \right]} \right]} \right)} \right)} }}$$

Where $\:{\:h}_{i}$is the feature vector of node $\:i$ (gene),$\:\:\:W$ is the learnable weight matrix, $\acute{a}$ is the learnable weight vector used in the attention mechanism, and $\:{\alpha\:}_{ij}\:$is the attention coefficient that tells how much node $\:j{\prime\:}\text{s}$ feature influences node $\:i$’s updated representation. The feature vector of the neighboring node $\:j\:\:\text{i}\text{s}\:{\:h}_{j}$, $\:\Vert\:\:$ indicates concatenation, $\:k$ represents neighboring nodes of node $\:i$, and $\:\:LeakyRelu$is nonlinear activation function.

After that, the aggregation process involves a linear transformation parametrized by a weight matrix W, followed by an attention operation. The attention operation assigns weights to each of the neighboring nodes based on the relevance of their features to the target node. Finally, the aggregated features are smoothed by an activation function $\:\left(\sigma\:\right)$, which adds non-linearity to the model. Aggregation function that combines features of all neighboring nodes $\:j$ of a given node $\:i$, where $\:{N}_{i}\:$is the neighbors of node $\:i$ can be defined by using (2)^30^:

2 $$\:{\:\stackrel{\prime }{h}}_{i}=\sigma\:{\sum\:}_{j\in\:{N}_{i}}{\alpha\:}_{ij}\:W{h}_{j}$$

$\:\:\:where\:\sigma\:$is the non-linear activation function.

**Fig. 3:**
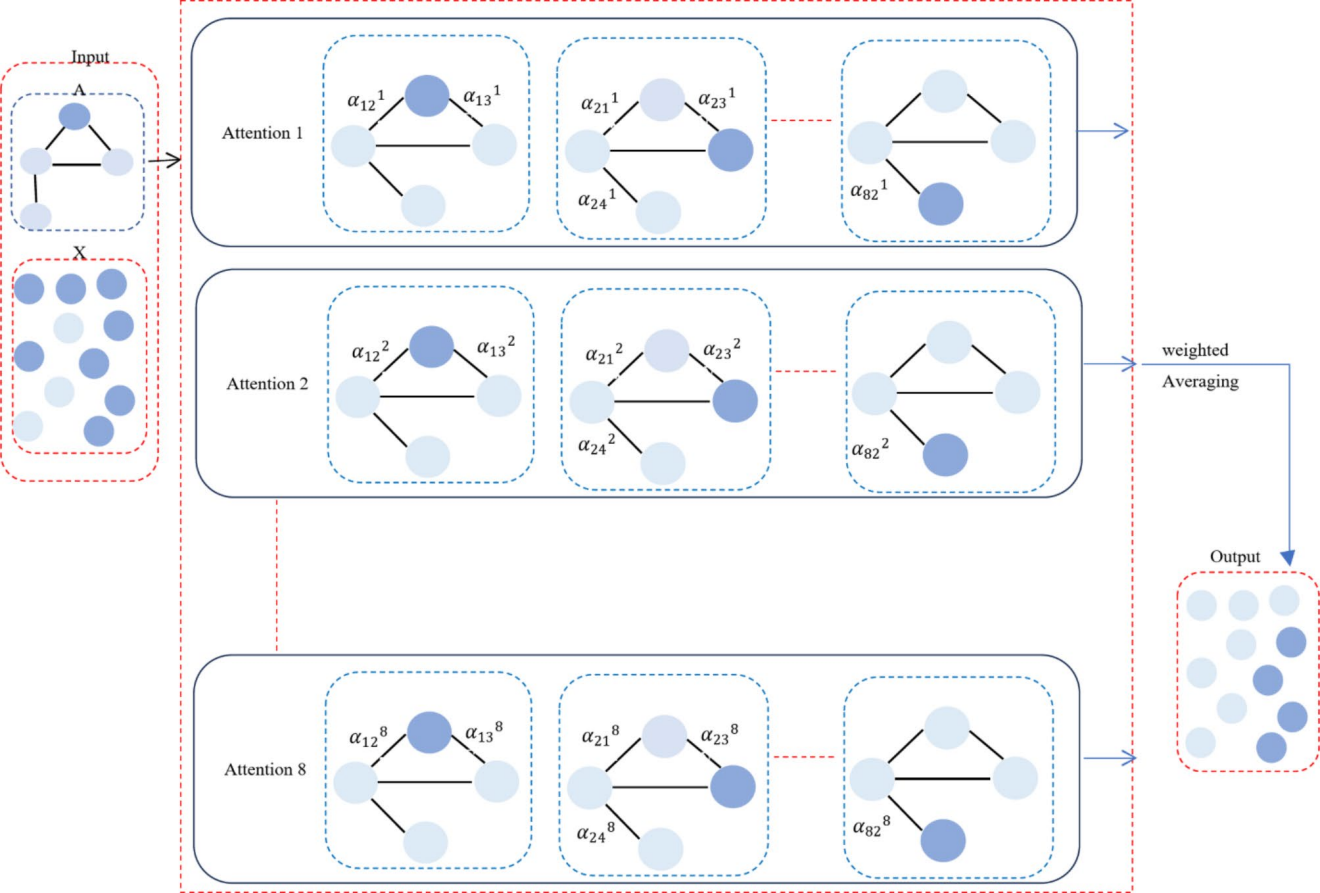
Multi-head attentions aggregation method in MSL-GAT.

To stabilize the learning process and improve model performance, GAT1 employs 8 multi-head attentions; in this approach, weighted averaging is a method of attention aggregation, as illustrated in Fig. 3. In this aggregation method, instead of treating all heads equally, you can weigh the outputs from each attention head differently, based on their importance or performance, as defined in (3). The weights can be learned during training or can be based on some criteria (performance on a validation set).

3 $$\:{\:\stackrel{\prime }{h}}_{i}\_after\:multiple\:head\:attentions=\sigma\:\left(\sum\:_{k=1}^{k}{W}_{k}{\sum\:}_{j\in\:{N}_{i}}{{\alpha\:}_{ij}}^{k}{W}_{k}{h}_{j}\right)$$

Where $\:{W}_{k}\:$is the weight assigned to the $\:{k}^{th}$attention head, $\:k$ is the number of attention heads. These weights can be learned during training or based on performance metrics, and $\:{\:\stackrel{\prime }{h}}_{i}$is the final node representation after the attention heads.

The weighted averaging method of aggregating multi-head attention outputs is more effective. It can allow the model to reduce noise, improve interpretability, and achieve adaptation to BL cancer heterogeneity, as BL cancer is so heterogeneous that fixed aggregation approaches are not effective. Learnable weights can be employed to adjust the model to the specific genetic landscape of every individual in order to address this. Non-coding RNA connections may be more significant for some patients than protein-coding gene interactions. This adaptability and customization increase the prediction’s accuracy. It also can boost the differential significance of gene interactions, as the PDG prediction pinpoints the essential genes for the development of cancer in certain patients. Weighted average sharpens the model’s emphasis on pertinent characteristics by giving greater weight to attention heads, which capture appealing, essential interactions. For example, if one attention head consistently produces more accurate or relevant features, you might assign it a higher weight when averaging the outputs. Weighted averaging allows the model to give more importance to the outputs from the attention heads that perform better, resulting in a more informed and potentially more accurate final feature representation. For example, assume you’ve trained the model and noticed that Head 1 consistently produces more accurate predictions or better features for tasks. If there are 8 attention heads, a weight of 0.5 must be applied to that head’s output and the remaining 0.5 must be distributed across the other heads.

EX: Outputs from four different attention heads:

- Head 1 output: [0.2, 0.4, 0.6]
- Head 2 output: [0.1, 0.5, 0.7]
- Head 3 output: [0.3, 0.3, 0.4]
- Head 4 output: [0.4, 0.2, 0.5]

However, since Head 1 is more accurate, you might want to give it more weight in the final aggregate. For instance, you could assign Head 1 a weight of 0.5 and distribute the remaining 0.5 across the other three heads. Let’s assume you assign the remaining heads a weight of ($\:\frac{0.5}{3}$ ) = 0.1667

First position: 0.5 × 0.2 + 0.1667 × (0.1 + 0.3 + 0.4) = 0.2333.

Second position: 0.5 × 0.4 + 0.1667 × (0.5 + 0.3 + 0.2) = 0.366.

Third position: 0.5 × 0.6 + 0.1667 × (0.7 + 0.4 + 0.5) = 0.566.

Updated node embeddings are the output of GAT1, which are features of every gene updated by attending to its neighbors. The GAT1 layer is followed by the $\:LeakyRelu$ layer, the batch normalization (BN) layer, and the dropout layer, which represent the activation and regularization technique. $\:LeakyRelu$ activation function after GAT1 to introduce non-linearity into the model, which enhances the network to learn complex aspects in the data. It enables some negative values to pass through, unlike the standard $\:Relu$ which sets all negative values to zero. Initial layers of the model are typically responsible for capturing more general patterns in the data. By applying $\:LeakyRelu$ after GAT1, firstly, network will handle negative information in genomic data. In BL cancer prediction based on PDG detection, some gene interactions or features may generate negative values after the GAT1 layer. These negative values may still hold useful information for prediction, so you don’t want to completely zero them out. $\:LeakyRelu$ ensures that negative information is retained, though with a reduced impact, allowing for a more flexible representation of the data. Secondly, improved gradient flow is achieved by allowing small negative values to propagate $\:LeakyRelu$, which ensures better gradient flow during backpropagation that can lead to more efficient training and potentially better performance in capturing complex patterns. For instance, assume that the output from $\:GAT1\:output=[1.2,\:-0.08,\:0.6,-0.4]$. After utilizing $\:LeakyRelu$ with (α = 0.01), $\:LeakyRelu\:output=[1.2,\:-0.008,\:0.6,-0.004]$. It keeps negative values with a small slope. This allows the network to continue updating weights for those negative activations.

By scaling and adjusting the inputs to have a mean of 0 and a standard deviation of 1, the BN layer standardizes the data entering a layer. As a result of the lessened internal covariate shift, training is stabilized and accelerated. By providing that the input distributions to each layer are normalized, BN enables the network to train more quickly and effectively. As a result, the network is more stable and less sensitive to weight initialization. Because gene expression data (both coding and non-coding genes) vary greatly, BN makes sure that each GAT layer’s input is on a steady scale, which speeds up training and improves network learning. It also improves the model’s ability to generalize to new data. A dropout layer is a regularization approach wherein during training, a portion of a layer’s neurons are arbitrarily “dropped” (set to zero). This promotes the model to acquire more robust, generic properties and keeps it from being overly dependent on any one neuron. When working with high-dimensional genomic data, such as that derived from coding and non-coding genes, dropout benefits in preventing overfitting. If the model picks up on the noise in the training set, it will become overfitted and perform poorly on untrained data. To make sure the model learns expansive correlations between genes rather than depending on particular patterns in the training data, dropout is used in the GAT layers after each attention layer.

The BN layer performs numerous vital duties when positioned after a GAT (Graph Attention Network) layer, particularly when handling both coding and non-coding genes. GAT models include complex computations and multi-layers, which can reach instability in training. Specifically, when exploding or vanishing gradients occur as they pass through layers.BN modifies and scales the activations to normalize the GAT layer’s output. By minimizing issues with exploding or vanishing gradients, this stabilization aids in maintaining a healthy gradient flow, allowing for more stable and rapid convergence throughout training. Internal covariate shift is the term used to describe the possibility of changes in the input distribution to each layer while training progresses. These modifications might hinder the model’s convergence and slow down the training process. By normalizing the inputs to the following layer to have a consistent distribution, BN reduces internal covariate shift. Because the parameters of each layer are not continuously changing in response to the changing input distributions, the model is able to learn more effectively^31^.

Higher learning rates without normalization might lead to the model diverging during training, particularly in complex structures such as those with several GAT layers. By maintaining that each GAT layer’s output has a stable mean and variance, BN enables the adoption of greater learning rates by reducing the sensitivity of the training process to initializations and enhancing its resilience to higher updates. Accurate predictions in PDG prediction depend on extracting pertinent information from a mixture of coding and non-coding genes. In order to improve understanding of the intricate relationships between genes, including the small distinctions between coding and non-coding genes in the prediction of BL cancer, batch normalization helps to refine the feature representations by ensuring that the inputs to the following layer are well-behaved (i.e., not too high or too low). BN ensures that activations keep within a range that is suitable to learning generalizable patterns, which minimizes overfitting in complicated models. In this task, where the model must operate effectively on unknown data from various patients, it also improves generalization across varied patient data^32^.

The dropout layer plays a beneficial role when placed after multi-GAT layers in the task of BL cancer prediction based on PDG, especially when working with both coding and non-coding genes. In complex approaches to GNN like GAT, which have multiple staked layers and multi-head attention mechanisms, a risk of overfitting issues may happen, especially when the MSL-GAT learns extremely particular patterns in the training dataset that do not generalize well to unknown data. The dropout layer is an ideal solution, which arbitrarily changes a portion of the input units at each training stage according to this task (set node features to zero). This forces the model to acquire more resilient, generalizable features that are not unnecessarily dependent on particular nodes or edges by preventing the model from depending too much on any one feature or attention head. According to the nature of genomic data, which is frequently high-dimensional and noisy, the GAT model is particularly vulnerable to learning noise in the data because of its intricate attention processes. Dropout achieves the regularization by comprising noise in the learning process; the model becomes more robust and improves its ability to generalize to new data. When analyzing genomic data, this is especially crucial because the aim is to identify significant driver genes that vary throughout individuals. Dropout can also boost the generalization concept. In the PDG prediction, since distinct instances of patients may have varied patterns of coding and non-coding gene expressions, the model must generalize effectively across these samples. The dropout layer helps the GAT to more equally spread the learned representation over all nodes by randomly removing nodes (and consequently the features associated with those nodes). This enhances the model’s generalization to novel, unknown patient data. The high-dimensionality of genomic data in GAT models can lead to the model learning erroneous associations, particularly in biological applications such as BL cancer gene prediction^33^. Dropout ensures that the features that the GAT layer learns are not unnecessarily particular to a single node or connection. This results in feature representations that are more resilient and valuable for a variety of samples and situational factors. The total output of GAT1 after applying activation and regularization techniques is the updated node embeddings for each gene after considering its neighbors, weighted by the attention mechanism. The output is passed to the next GAT layer, as determined by )4(.

4 $$\:{\:\:Total\_h}_{1}\:=Dropout\left(BN\right(Leakyrelu\:\left(GAT1\right(node\_features,\:\:adjacency\:\_matrix\left)\right))$$

Where $\:{\:Total\_\:h}_{1\:\:}$is the output of GAT layer 1 It represents the updated node embeddings after applying the first graph attention layer.

#### GAT layer 2 with (residual connection)

Total output ($\:{\:\:Total\_h}_{1})$ of GAT1 is an input of GAT2. GAT2 layer processes $\:{\:\:Total\_h}_{1}$ through eight multi-head attention. Then pass it through $\:Leakyrelu$, the output of GAT2 can be represented as $\:{\:\:Total\_h}_{2}$, as calculated in (5):

5 $$\:{\:\:Total\_h}_{2}\:=\:Leakyrelu\:\left(GAT2\right({\:\:Total\_h}_{1},\:\:\:adjacency\:\_matrix\left)\right)$$

Where $\:{\:Total\_\:h}_{2\:\:}$is the output of GAT layer 2 that passes through the nonlinear activation function layer. It represents the updated node embeddings after applying the second graph attention layer.

A residual connection is applied between GAT1 and GAT2. The output of GAT1 is fed directly to the residual connection and then integrated with the $\:{\:\:Total\_h}_{2}$ before passing it through BN and dropout, followed by feeding it into GAT3. This makes sure that GAT3 can keep on using the original features from GAT1 even if GAT2 doesn’t pick up any major modifications. This structure handles prospective issues, including overfitting and vanishing gradients, while facilitating efficient feature propagation. By immediately transferring the original output of GAT1 ($\:{\:\:Total\_h}_{1}$ )to the input of the subsequent layer, the model improves the flow of gradients during backpropagation, consequently simplifying the training of deep networks. The position of the residual connection between GAT layer 1 and GAT layer 2 is based on many reasons. This can accomplish stability of learning in the early layers. Residual connections are vital in early layers of this approach, where the model learns essential features^31,32^. In MSL-GAT, GAT layer 1 picks up initial gene relationships, which are critical for further processing. Position of residual connection maintains these early and basic features, which are frequently low-level or general, even as layers capture more intricate relationships.

The placement of residual connections enables the model to prevent degradation in early layers. Early or primary layers in deep methods, especially when processing complicated data like genomic data, can endure gradient vanishing challenges, where gradients become very tiny, resulting in ineffective learning. The residual connection assists to avoid this by allowing the gradient a shortcut to pass through, boosting Layer 2 learning. At this point, a residual connection may add noise or low-level aspects that are no longer required if Layer 2 is already learning extremely abstract relationships. Because Layer 3 usually focuses on high-level abstractions, it is therefore often less beneficial to apply a residual connection between Layer 2 and Layer 3. The output of the residual connection is defined by using (6):

6 $$\:{\:\:\:h}_{residual}\:=\:{\:Total\_\:h}_{1+}{\:Total\_\:h}_{2\:}$$

Where $\:{\:\:\:h}_{residual}\:$is residual connection output.

After adding the residual connection, apply BN and dropout to the result to stabilize and regularize the network. The final output is calculated by using (7):

7 $$\:{\:\:h}_{final}=\:Dropout\left(BN\left({\:\:h}_{residual}\right)\right)$$

Where $\:{\:\:\:h}_{final}\:$is the final output of GAT layer 2 after passing $\:{\:\:\:h}_{residual}\:$through $\:\:Dropout\:and\:BN\:layers$

#### GAT layer 3 and three dense blocks

The $\:{\:\:h}_{final}$ is fed into GAT3 for further feature extraction. It is also utilized for eight multiple-head attentions. It is followed by $\:Leaky\:relu$ activation function, BN, and dropout. After activation, batch normalization, and dropout. GAT3 further refines the features, capturing higher-order dependencies in the graph. GAT3 produces an accumulation of node embeddings that capture the graph’s acquired features, as defined by using (8).

8 $$\:{\:\:Total\_h}_{3}\:=\:Dropout\left(BN\right(Leakyrelu\:\left(GAT3\right({\:\:h}_{final},\:\:\:adjacency\:\_matrix\left)\right))$$

Where $\:{\:\:Total\_h}_{3}\:\:$is the output of GAT layer 3, It represents the updated node embeddings after applying the third graph attention layer.

The output of GAT3 (after the activation, batch normalization, and dropout) is a set of node embeddings that encapsulate the learned features from the graph. This output can be fed directly into dense blocks for further processing, which is a classification task, to give a final prediction of whether each patient is at risk of being injured with BL cancer or not. In the task of BL cancer prediction based on PDGs (coding and non-coding), the decision to utilize multi-dense blocks or just only one after using the three GAT layers is based on the feature relationship complexity deduced from the GAT layers that multiple dense blocks can be beneficial in forecasting delicate characteristics from GAT layers. It needs another non-linear transformation before reaching the last prediction. Deeper feature transformation shows that multiple dense blocks are helpful for complicated non-linear patterns such as gene interactions in the prediction process since they conduct deeper adaptations and improve feature representations. Achieving more flexibility is required because more dense blocks can lead the model to get more flexibility to pick up complex interactions and relationships in the embeddings^34^.

**Fig. 4:**
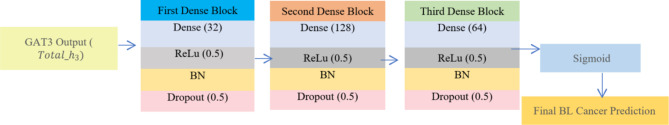
The structure of three dense blocks of MSL-GAT.

The choice of three dense blocks, as illustrated in Fig. 4, relies on many reasons, like: (i) Feature refinement and non-linear transformations: The dense blocks have been designed to further refine the node embeddings provided by the GAT layers. While GAT layers are very effective in capturing the relationship among nodes based on graph topology and attention mechanisms, the dense blocks transform these high-dimensional embeddings into more suitable representations for the binary classification task, namely, predicting PDGs in BL cancer. The first dense block amplifies the high-level features learned by the GAT layers, while the second one reduces feature dimensionality by focusing on the most relevant features to prevent overfitting. The third dense block then takes this learned representation, aggregates it, and fine-tunes it to predict the final predictions. (ii) Overfitting and over smoothing: Having only one or two dense blocks might result in underfitting of the model by failing to capture important nonlinear relationships among the data. On the other hand, using more than three blocks may overfit or be computationally unnecessary for high-dimensional genomic data. The use of three dense blocks balances performance with complexity. (iii) Depth and task complexity: PDGs prediction from coding and non-coding genomic data is a complex task that embeds highly heterogeneous features and intricate interactions. The three dense blocks have enough capacity to model such complexities without adding redundancy or overcomplicating the architecture. (iv) Compatibility with GAT Outputs: Basically, embeddings from GAT layers are meant to capture graph-based relationships, which require multiple levels of dense transformations in order to finally integrate with the classification head. These dense layers align the graph-based features with the classification objective.

Dense blocks (also called fully connected layers) are essential components that enable a neural network to learn and capture more complex patterns by integrating the retrieved feature embeddings from the prior layers (in this task, the GAT layers). MSL-GAT output is an accumulation of node embeddings that show relationships and picked-up features from the graph (genomic data). Dense layers subsequently analyze these embeddings to complete the ultimate prediction task of BL cancer^33,34^. Each dense block frequently consists of major components, which are the dense layer, activation function, BN layer, and finally, the dropout layer. A dense layer is a fully connected layer that receives the input from the prior layer and converts it into an output of the appropriate size. Every neuron in this layer is associated with each neuron in the previous layer. Activation After the dense layer is implemented to enable the model to capture more elaborate patterns, the BN layer is usually utilized after the activation function to make sure that the results of the dense layer are normalized. This assists the approach to train quickly and enhance generalization. Each layer’s output is standardized by BN through subtracting the batch mean and dividing the result by the batch standard deviation. During training, dropout is used to reduce overfitting by randomly changing a portion of the neurons to 0. This motivates the model to select more reliable features. In the last layer, sigmoid activation is used to predict BL cancer (for binary classification). The first dense block receives the output from the third GAT layer (GAT3). The intent of the first dense block is to boost the features that the GAT layers retrieved. The first dense block functions as a non-linear transformation layer that may learn intricate relationships and interactions between these high-level attributes since the GAT layers are primarily concerned with capturing complicated node-level connections and attention procedures. To the second dense block, the output from the first dense block is passed on. Through feature compression into a more condensed structure, the second block usually lessens the dimension of the features. By restricting the model’s features and concentrating on the most crucial characteristics for the given target (PDG prediction), this helps to lessen overfitting. The last dense block receives its output from the second dense block^34^. This block’s main responsibility is to generate the final prediction. This block would apply to the proper activation function (sigmoid) depending on the classification task (e.g., identifying if a gene is a driver gene or not), then give the final decision of BL cancer prediction of individualized patients.

## Experimental results

This section will describe the execution of the proposed MSLGAT, which utilizes a new structure of GAT that consists of three layers. Each one can play a crucial role in predicting BL cancer based on PDGs. This novel constructure of GAT, which can deal with a heterogeneous genomic dataset (coding and non-coding genes), comprises three dense blocks. After implementing the MSL-GAT to capture the most vital features of genes and complex relationships, the fully connected layers can serve as essential components for refining the learned representations from the GAT layers. The recommended approach will be executed in an essential scenario. This scenario is applying the suggested MSL-GAT to the TCGA-BLCA dataset and contrasting it with other contemporary methodologies. Our implementation is predicated on a genomic dataset of coding and non-coding genes. The accuracy, precision, recall, and F1-score measures will be utilized to gauge the MSL-GATS’s efficacy.

### The description of BL cancer genomic dataset

This study contains all BL cancer cases from The Cancer Genome Atlas (TCGA), which is an open-access database managed by the National Cancer Institute and the National Human Genome Research Institute. Since 2006, approximately 20,000 primary cancer and matched normal cases from 33 various kinds of cancer have been identified and stored in the collection^35^. The BLCA dataset, which includes both coding and non-coding genes, is the most comprehensive dataset. This investigation relies on the TCGA-BLCA dataset solely because of both appropriate and effective reasons, as follows: (i) High-quality and extensive dataset: One well-known and reliable dataset that supplies outstanding, meticulously chosen genomic and clinical data for BL cancer is TCGA-BLCA. A wide range of patient data, including multi-omics statistics and knowledge (such as methylation, RNA-Seq, CNVs, and somatic mutations), are included. By training our model on intricate physiologically significant variables, the dataset offers an extensive overview of the molecular landscape of bladder cancer, improving its prediction performance. (ii) Sufficient patient diversity: A model that can generalize to many patient scenarios may be developed due to the TCGA-BLCA dataset, which provides an exhaustive cohort of patients that reflects the variety of bladder cancer across populations. (iii) Reproductivity in genomic studies: Due to their direct comparison with other methods and repeatability, TCGA datasets are often utilized in cancer genomics research, allowing researchers to confirm and expand on their findings. (iv) The model underwent rigorous validation techniques, including cross-validation and sub-cohort analysis, to ensure its robustness and generalizability across various clinical and molecular characteristics. (v) External dataset issues: Fair comparisons may be complicated by the lack of comprehensive genomic feature integration and extensive annotations in external databases for BL cancer assessment. This dataset specifically presents: (i) Coding Genes: The TCGA-BLCA dataset includes whole-exome sequencing (WES) and RNA-Seq data on the genome’s coding regions. (ii) Non-Coding Genes: RNA-Seq data, which quantifies the expression of non-coding RNAs, such as long non-coding RNAs and microRNAs, is also gathered. DNA methylation data in the TCGA-BLCA dataset may also contain regulatory elements in non-coding regions. As a result, studies that aim to identify unique driver genes in bladder cancer by combining coding and non-coding genomic information could find the TCGA-BLCA dataset particularly suitable. This dataset is accessible via the Genomic Data Commons (GDC) Data Portal. For predicting BL cancer and detecting personalized driver genes, the following data file types are needed: (ii) Somatic Mutation Data (ii) MAF Files: includes data on mutations in coding and non-coding regions. (iii) RNASeq Data: Gene expression profiles for both coding and non-coding RNAs. (iv) Copy Number Variations (CNVs): presents information on genomic amplifications and deletions. (v)DNA Methylation Data: contains information on epigenetic modifications. To achieve optimal model performance, all labeled genes are split, using 10% as the test set, 10% as the validation set, and 80% as the training set. Experimental code is implemented based on the open-source machine learning framework TensorFlow. For your MSLGAT structure with three layers, BN, a residual connection, a dropout rate of 0.5, (α = 0.01), and no. epochs = 200.

### Performance metrics for MSLGAT

In this section, the hypothesized model, known as MSLGAT, for predicting BL cancer will be assessed with a variety of metrics, all of which will be supplied. Deep GCN algorithms are often evaluated using metrics like F1-score, accuracy, recall, and precision. The values of the variables false positive (FP), false negative (FN), true positive (TP), and true negative (TN) are obtained from the confusion matrix. TP are the output values that are initially positive and are anticipated as positive outputs by the model. The assessment metrics—precision, recall, accuracy, and F1-score—are essential for evaluating a predictive model’s performance in BL cancer prediction. Various features of the model’s efficacy for forecasting cancer cases are shown by these measures (e.g., recognizing individuals with BL cancer from a dataset). Accuracy can be measured by the proportion of all correct predictions (both true positives and true negatives) out of the total number of predictions made by using (9). Sensitivity, or recall, can be calculated by the proportion of actual bladder cancer cases that were correctly identified by the model using (10). Also, by the proportion of true positive predictions (i.e., correctly predicted bladder cancer cases) out of all positive predictions made by the model, precision may be computed using (11). Using (12), the F1-score may be computed as a sum of recall and accuracy.

9 $$\:Accuracy=\frac{TP+TN}{\left(TP+TN+FP+FN\right)}\:\:\:\:$$

10 $$\:Recall=\frac{TP}{\left(TP+FN\right)}$$

11 $$\:\text{P}\text{r}\text{e}\text{c}\text{i}\text{s}\text{i}\text{o}\text{n}=\frac{TP}{\left(TP+FP\right)}$$

12 $$\:F1-score=2\text{*}\left(\frac{Precision\text{*}Recall}{Precision+Recall}\right)$$

To evaluate the performance of the MSL-GAT approach for BL cancer prediction based on the identification of PDGs, TCGA-BLCA was used as a basic benchmark. The top 100 genes predicted by various approaches were chosen, and the performance of these methods was evaluated using three metrics: precision (P), recall (R), and the F1-score. The proportion of successfully predicted partial driver genes among the TCGA-BLCA driver genes is denoted by (R), and the fraction of correctly predicted driver genes among the predicted driver genes is denoted by (P). The ability to identify cancer driver genes on the TCGA-BLCA database may be efficiently evaluated using the F1-score, a statistic that combines precision and recall.

### Testing the multi-stacked layered GAT strategy (MSL-GAT) against other strategies

**Fig. 5:**
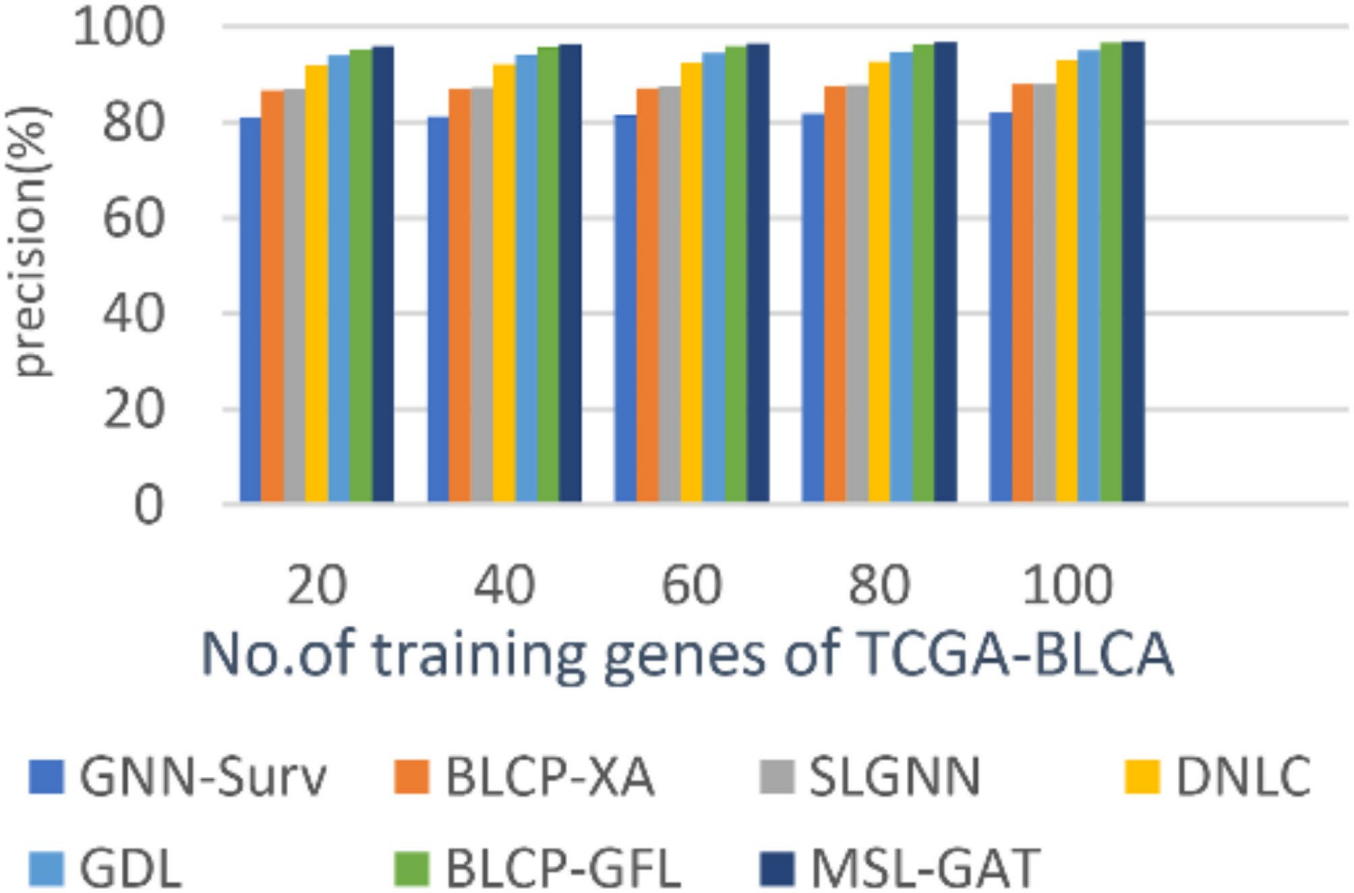
Precision of MSL-GAT.

**Fig. 6:**
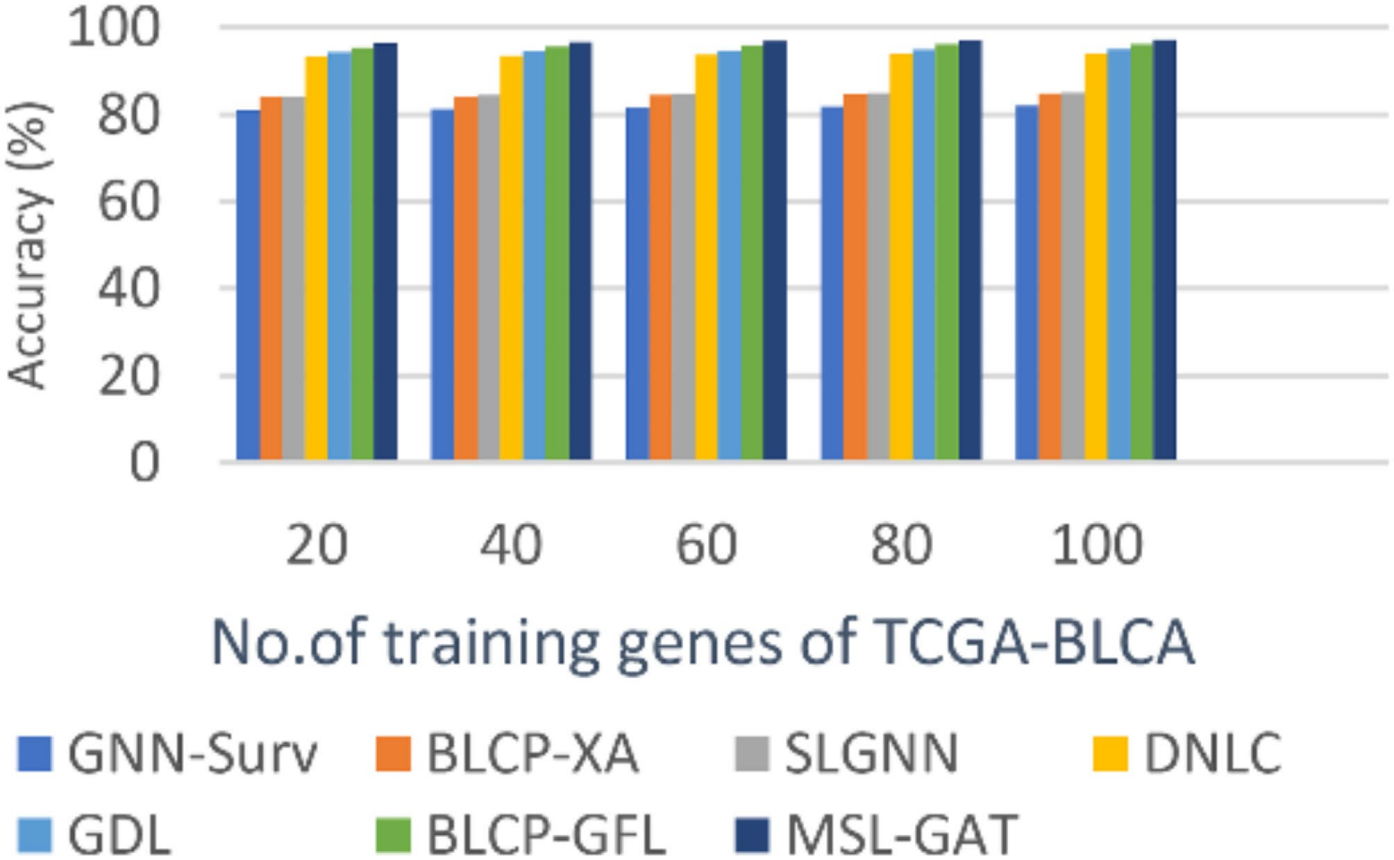
Accuracy of MSL-GAT.

**Fig. 7:**
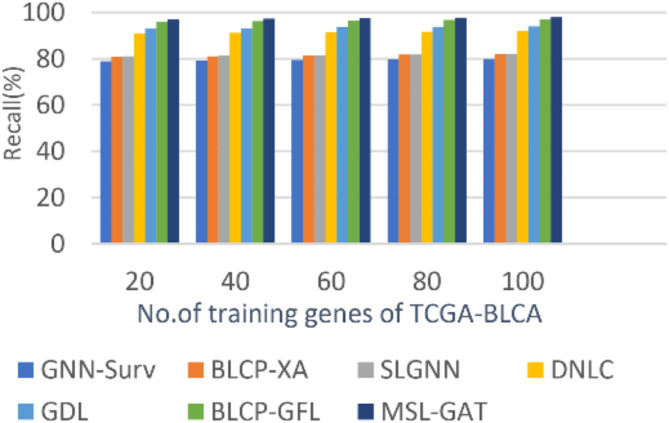
Recall of MSL-GAT.

**Fig. 8:**
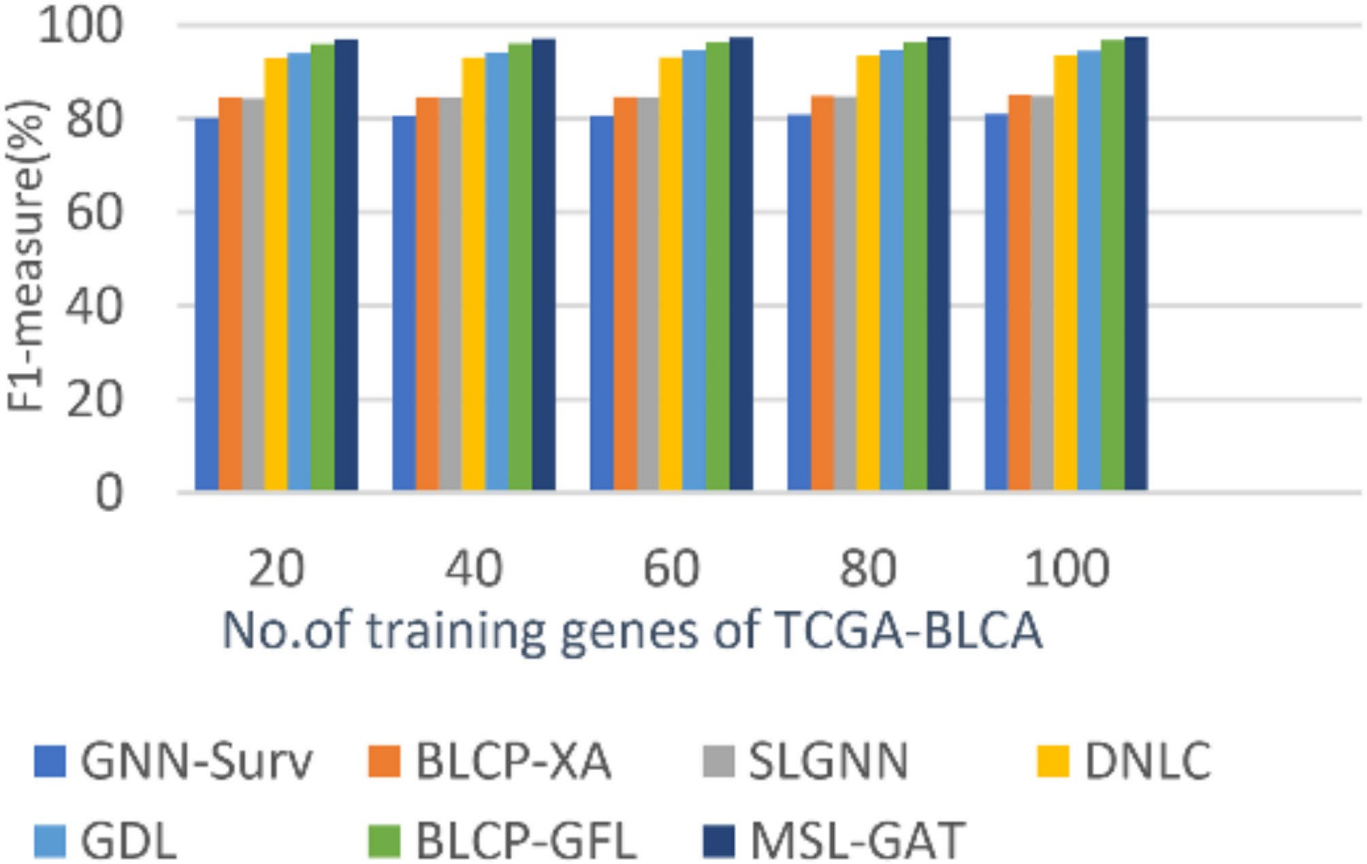
F1-measurel of MSL-GAT.

In this subsection, the BL cancer prediction method, which is called MSL-GAT, can be demonstrated by testing and comparing it with other contemporary prediction methods to demonstrate its efficacy. These other prediction approaches are BLCP-GAFR^12^, BLCP-XA^13^, GNN-Surv^14^, SLGNN^15^, DNLC^16^, and GDL^17^. Figures 5, 6, 7 and 8; Table 1 illustrate the accuracy, precision, recall, and F1-score of the BL cancer prediction strategies. As the suggested MSL-GAT may yield the best performance results, it really performs better than other BL cancer prognostic techniques. MSL-GAT offers the greatest values of accuracy, precision, recall, and F1-score at a number of training instances = 100, corresponding to 97.72%, 97%, 97.98%, and 97.487%, respectively. The whole result varied when the number of dense blocks was modified, as illustrated in Table 1, which reflects the role of dense blocks in prediction results, and its number choices are a crucial decision.

**Table 1:** MSL-GAT performance metrics with different numbers of dense blocks.

Metrics	MSL-GAT (With 1Dense Block)	MSL-GAT (With 2Dense Blocks)	MSL-GAT (With 3Dense Blocks)	MSL-GAT (With 4Dense Blocks)
Accuracy (%)	95.45	96.58	97.72	97.60
Precision (%)	94.50	96.00	97.00	96.75
Recall (%)	94.85	96.40	97.98	97.30
F1-Score (%)	94.67	96.20	97.49	97.02

Figures 5, 6, 7 and 8; Table 2 show that MSL-GAT surpasses BLCP-GAFR, BLCP-XA, GNN-Surv, SLGNN, DNLC, and GDL. Conversely, the accuracy values for numerous training instances = 100 for BLCP-GAFR, BLCP-XA, GNN-Surv, SLGNN, DNLC, and GDL are 96.1%, 84.89%, 82%, 85%, 94%, and 82%, while their precision values are 96.66%, 88%, 82%, 88%, 93%, and 95%, respectively. Their values in other performance metrics like recall at the number of training cases = 100 are 96.889%, 81.53%, 80%, 82%, 92%, and 94%, while their values for F1-score are 96.774%, 84.62%, 80.987%, 84.895%, 93.5%, and 94.49%, respectively. Therefore, the GNN-Surv approach yields the lowest accuracy, precision, recall, and F1-score values, whereas the MSL-GAT technique yields the highest values in those areas. Furthermore, BLCP-GAFR is the second-best approach in terms of precision and recall values, while GDL is the third-best approach. As a result, DNLC came in fourth, SLGNN came in fifth, and BLCP-XA came in sixth. The best technique is represented by MSL-GAT, while the poorest strategy is GNN-Surv.

**Table 2:** BL Cancer prediction performance metrics at the number of training instances = 100.

Bladder cancer (BL) prediction Methods	Accuracy	Precision	Recall	F1-score
GNN-Surv	82	82	80	80.987
BLCP-XA	84.89	88	81.53	84.62
SLGNN	85	88	82	84.895
DNLC	94	93	92	93.5
GDL	95	95	94	94.49
BLCP-GAFR	96.1	96.66	96.889	96.774
MSL-GAT	97.72	97	97.98	97.4875

MSL-GAT shows the highest overall performance with the best accuracy, recall, and F1-score, making it suitable for clinical settings. BLCP-GAFR ranked second as it stands out due to its focus on explainable techniques and interpretable outputs. BLCP-GAFR achieves high prediction accuracy (96.1%) with interpretable rules and validates selected genes for relevance to BL cancer pathways. Two gene analysis techniques are MSL-GAT and BLCP-GAFR. While BLCP-GAFR employs a genetic algorithm and fuzzy rule-based system for transparent decision-making, offering a full perspective of gene regulation, MSL-GAT uses multi-stacked layers of GAT, followed by dense blocks for extensive analysis of gene relationships. Because of its rule-based categorization, BLCP-GAFR is easier to comprehend, but MSL-GAT is more flexible in response to genetic changes. Whereas BLCP-GAFR concentrates on methylation and differential expression, MSL-GAT manages both coding and noncoding genes. Additionally, BLCP-GAFR depends on microarray data, which could not accurately represent gene regulation. Multiple explainability strategies make it difficult for clinical usage and lack external validation.

The GDL model ranked third with 95% accuracy and 94% recall. By combining coding and non-coding genes, MSL-GAT offers a more thorough examination of the genome. As a result, it is more appropriate for recording intricate relationships over the whole genome. GDL, on the other hand, is mostly concerned with exonic mutations (coding areas). This restricts its capacity to identify regulatory components in non-coding areas, which may cause it to overlook certain important indicators of the advancement of bladder cancer. MSL-GAT is appropriate for evaluating customized driver genes because of its graph-based design, which enables it to record gene associations. It is quite good at comprehending how things operate inside a network framework. Although GDL’s deep neural network is excellent at quickly detecting mutations unique to cancer, it lacks the relational insight that graph structures offer. Because MSL-GAT is graph-based, it provides greater versatility.

There are two distinct methods for examining gene interactions: MSL-GAT and DNLC. Whereas DNLC employs a deep neural network architecture, MSL-GAT uses a graph-based design to capture gene interactions. Because MSL-GAT incorporates both coding and non-coding genes, it is more appropriate for examining intricate genomic interactions. However, DNLC does not explicitly describe gene interactions; instead, it concentrates on feature selection and prediction utilizing genomic and clinical data. Because of its high computing resource requirements and computational intensity, DNLC is best suited for settings with strong computational infrastructure. With an emphasis on locating important characteristics and markers in both coding and non-coding areas, DNLC is highly effective at predicting cancer in its early stages. With an emphasis on driver genes (PDGs) that affect patient-specific outcomes, MSL-GAT finds and analyzes genes that directly affect BL cancer. It improves the interpretation of genomic data and offers thorough insights by enabling the model to incorporate gene interactions at various sizes. With an emphasis on driver genes (PDGs) that affect patient-specific outcomes, MSL-GAT performs better than DNLC in identifying and prioritizing genes that directly affect BL cancer. It improves the interpretation of genomic data and offers thorough insights by enabling the model to incorporate gene interactions at various sizes.

MSL-GAT enhances tailored driver gene discovery by capturing both local and global genomic interactions at many scales. Despite its interpretability issues, SLGNN uses knowledge graphs to produce more structured predictions. Although this method provides more precise insights into gene connections, further research is still needed to properly understand it for doctors. SLGNN and MSL-GAT both have significant processing needs; however, because of its multi-scale graph attention structure, MSL-GAT uses more resources. The complexity of maintaining and integrating knowledge graphs presents scaling issues for SLGNN. SLGNN may have less generalizability because of the availability of inconsistent high-quality SL gene data across cancer datasets, but MSL-GAT provides greater flexibility to other datasets because of its emphasis on larger genomic connections.

The MSL-GAT model is appropriate for thorough genomic analysis and individualized cancer prediction because it uses dense blocks in conjunction with multi-scale graph attention to capture both local and global interactions between coding and non-coding genes. In contrast, BLCP-XA analyzes gene expression data utilizing a variety of interpretable AI approaches, with the primary goal of finding important genes in bladder cancer through results that are clear and understandable. MSL-GAT combines multi-scale analysis and dense feature propagation to maximize prediction accuracy. Because BLCP-XA places a high value on interpretability, its predictions are simpler to comprehend and more useful to physicians. However, when compared to more sophisticated models like MSL-GAT, this could result in worse accuracy performance. By combining coding and non-coding genes, the MSL-GAT method may be adjusted to fit more extensive genomic data. On the other hand, BLCP-XA can only analyze microarray data and concentrates on gene expression rather than a comprehensive genomic picture, which may limit its capacity to adapt to other kinds of data. While BLCP-XA is more dataset-specific and might not transfer well to other datasets or patient groups without further validation, MSL-GAT is more adaptable and capable of handling a variety of data types.

Based on both coding and non-coding genomic data, MSL-GAT is primarily intended for prediction, with a particular emphasis on discovering and forecasting customized driver genes in bladder cancer. Conversely, GNN-Surv uses patient similarity networks to forecast survival outcomes based on clinical and genetic data in order to predict survival. By combining coding and non-coding gene interactions, MSL-GAT analyzes genomic data to pinpoint important factors that influence the development of bladder cancer. GNN-Surv is more focused on patient outcome prediction and has a wider range of data modalities since it integrates genetic and clinical data to create patient similarity networks that allow survival predictions. Layers of complexity are added by MSL-GAT with dense blocks to enhance feature representation and extraction. Even so, its predicted accuracy is better. The combination of several survival models and patient similarity networks makes GNN-Surv’s implementation complicated, but its emphasis on survival prediction gives it a clear edge in clinical prognostic situations. Predictive power is given priority by MSL-GAT, which focuses on the interpretability of multi-scale genomic interactions to achieve improved classification accuracy. Despite providing better survival prediction accuracy, GNN-Surv has interpretability issues since GNNs often give little information on how certain factors affect predictions. Due to its greater adaptability to various genomic datasets, MSL-GAT may be used for a wider range of genomic studies and personalized medicine applications. It is difficult and calls for in-depth understanding of graph theory, machine learning, and survival analysis to integrate patient similarity networks, GNNs, and survival models in GNN-Surv. The success of the model depends on the quality of the data, and performance may be impacted by missing or inconsistent data. Because of its deep learning architecture, GNN-Surv also has reduced interpretability, which makes it challenging to give doctors clear insights.

#### External validation and robustness

**Fig. 9:**
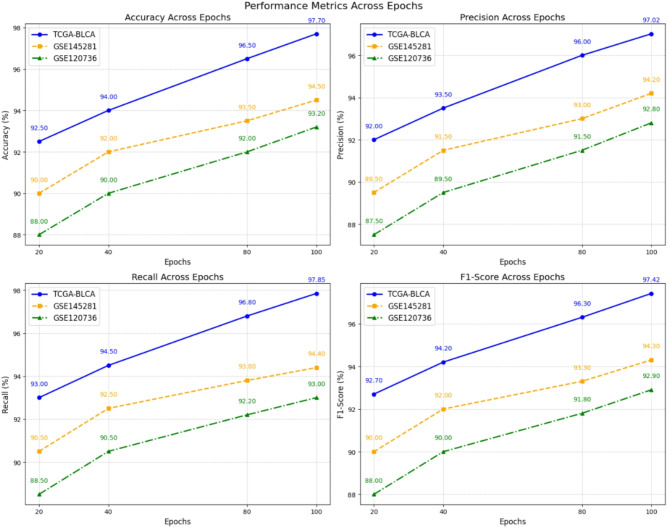
MSL-GAT Validation with New Dataset.

To further assess the robustness and generalizability of our model, we implemented external validation on independent datasets such as GSE145281. It is single-cell RNA sequencing data of peripheral blood mononuclear cells (PBMCs) from bladder cancer patients treated with atezolizumab (anti-PD-L1 mAb) in a Phase II trial, IMvigor210. The dataset is valuable for understanding immune cell gene expression profiles that characterize treatment response. GSE120736 is Containing gene expression profiles from 118 primary bladder cancer samples and 27 recurrent bladder tumor tissues, this dataset facilitates the identification of molecular subtypes and biomarkers associated with clinical outcomes. Results, summarized in Table 3, indicate that the MSL-GAT model maintains high performance on external datasets with accuracy, precision, recall, and F1-score close to those obtained on TCGA-BLCA. These results demonstrate the robustness and generalizability of our model, addressing concerns about overfitting and highlighting its potential for clinical applications, as illustrated in Fig. 9.

**Table 3:** MSL-GAT external validation with independent dataset.

Metrics	TCGA-BLCA	GSE145281	GSE120736
Accuracy (%)	97.7	94.5	93.2
Precision (%)	97.02	94.2	92.8
Recall (%)	97.85	94.4	93
F1-Score (%)	97.42	94.3	92.9

#### Biological and clinical relevance of identified driver genes

The MSL-GAT model identified key driver genes implicated in bladder cancer biology, including TP53, FGFR3, and CDKN2A, which play essential roles in tumor suppression, cell cycle regulation, and oncogenic signaling. Additionally, the model predicted non-coding elements such as HOTAIR and MALAT1, which are known to contribute to metastasis and drug resistance. To investigate the biological significance of these genes, pathway enrichment analysis was performed using Reactome and Wiki Pathways, two publicly available and widely validated databases. The analysis revealed strong associations with pathways such as PI3K-Akt signaling, p53 signaling, and cell cycle regulation, all of which play a critical role in bladder cancer initiation, progression, and therapeutic resistance. The p53 signaling pathway, for example, is frequently disrupted in muscle-invasive bladder cancer (MIBC) due to TP53 mutations, leading to uncontrolled cell proliferation and genomic instability. Similarly, PI3K-Akt signaling is hyperactivated in bladder tumors, promoting tumor growth, angiogenesis, and resistance to apoptosis. These pathway associations provide biological context for MSL-GAT’s predictions, demonstrating their potential role in early diagnosis and personalized therapeutic interventions. To further validate the clinical significance of the predicted driver genes (PDGs), we cross-referenced them with COSMIC and OncoKB, two established cancer gene databases. The MSL-GAT model consistently identified well-known bladder cancer driver genes such as TP53, FGFR3, and CDKN2A, reinforcing the model’s robustness and biological relevance. Additionally, our findings highlighted non-coding RNAs like HOTAIR and MALAT1, whose roles in cancer progression and therapy resistance have been previously documented. The identification of these lncRNAs as potential biomarkers underscores the model’s capacity to uncover novel regulatory mechanisms in tumor biology^36,37^.

The functional impact of the identified genes was further analyzed through Reactome and Wiki Pathways, ensuring their clinical and molecular relevance. The results suggest that mutations or copy number variations (CNVs) in TP53 and FGFR3 may correlate with aggressive bladder cancer subtypes, making them valuable prognostic markers. Similarly, the identified lncRNAs could serve as potential therapeutic targets, opening avenues for RNA-based therapies, such as RNA silencing or antisense oligonucleotides. Unlike traditional graph-based learning models such as GDL, which primarily rely on pathway-level associations, MSL-GAT integrates both coding and non-coding genomic features, offering a comprehensive and interpretable view of tumor biology. This integrative approach enhances biomarker discovery, improves predictive accuracy, and provides biologically meaningful insights that are crucial for translational cancer research. By leveraging open-access pathway databases, the model ensures that findings remain transparent, reproducible, and clinically relevant.

## Conclusions and future works

This paper presents a deep graph convolutional neural network strategy-based attention mechanism for BL cancer prediction called MSL-GAT. This strategy used the TCGA-BLCA dataset, which contains both coding and non-coding genes. Coding genes are directly responsible for producing proteins; non-coding genes, such as long non-coding RNAs (lncRNAs), also play critical roles in regulating gene expression, tumorigenesis, and cancer prediction and progression. The proposed approach, MSL-GAT, comprises three stacked layers of GAT attached with multi-regularization techniques like BN and dropout to focus on the most relevant genes (nodes) and relationships (edges) in the patient-specific genomic interaction network (PPI). Three GAT layers are followed by three dense blocks because of model complexity, feature extraction, computational resources, and the size of the dataset. A balance is needed to ensure the proposed model is powerful enough to learn the complex interactions in genomic data but also interpretable and efficient. Finally, BL cancer prediction is obtained from the last activation layer (sigmoid function). The MSL-GAT achieves the best results in accuracy, precision, recall, and F1-score. The final results illustrate that the accuracy of MSL-GAT is 97.72% for the BL cancer prediction task.

In the future, this strategy may combine with temporal dynamics by adding a temporal component to model changes in gene expression over time (using techniques like LSTM or GRU layers). Integrating temporal components with LSTM or GRU layers after GAT layers can accurately predict coding and non-coding gene changes at different cancer stages, especially for personalized driver gene prediction, affecting treatment outcomes. Applying transfer learning or pre-trained graph neural network (GNN) models to leverage prior knowledge from other cancer types or diseases. This approach could help overcome the limited availability of bladder cancer-specific data by borrowing knowledge from similar datasets (e.g., other urological cancers). Implement ensemble learning techniques by combining the GAT-based model with other machine learning models such as random forests, SVMs, or CNNs to improve the robustness and accuracy of predictions by combining the strengths of multiple models. Although the present investigation has concentrated on TCGA-BLCA, we want to broaden our validation to other datasets (such as the METABRIC and GEO datasets) in further research. This will further illustrate our model’s generalizability.

## Data Availability

The dataset supporting the results of this study is publicly available at the Genomic Data Commons (GDC) repository: https://portal.gdc.cancer.gov/. All relevant data can be accessed under the TCGA-BLCA project. The data used from TCGA-BLCA is publicly available, and all patient information has already been anonymized and de-identified by TCGA, adhering to ethical guidelines. Additionally, the GSE145281 dataset is publicly available at the NCBI Gene Expression Omnibus (GEO) and can be accessed at: https://www.ncbi.nlm.nih.gov/geo/query/acc.cgi?acc=GSE145281. All patient information associated with this dataset has been anonymized and de-identified, adhering to ethical guidelines. The dataset supporting the results of this study is publicly available. The GSE120736 dataset, which includes comprehensive molecular classification data for bladder cancer, is available in the NCBI Gene Expression Omnibus (GEO) and can be accessed at: https://www.ncbi.nlm.nih.gov/geo/query/acc.cgi?acc=GSE120736. All patient information associated with this dataset has been anonymized and de-identified, adhering to ethical guidelines. For pathway enrichment analysis, Reactome and Wiki Pathways, two publicly available and widely used open-access pathway databases, were utilized. The pathway data can be accessed at: Reactome Pathways (e.g., PI3K-Akt Signaling Pathway, p53 Signaling Pathway) PI3K-Akt Signaling Pathway: R-HSA-199420 (https://reactome.org/PathwayBrowser/#/R-HSA-199420), p53 Signaling Pathway: R-HSA-69541 (https://reactome.org/PathwayBrowser/#/R-HSA-69541), Wiki Pathways (e.g., Bladder Cancer Pathways), Bladder Cancer Pathway: WP2821 (https://www.wikipathways.org/instance/WP2821) Both databases provide freely accessible, curated pathway information to ensure transparency, reproducibility, and proper attribution. No special permissions are required for their academic use.

## References

[1] Zhang, F., Geng, J., Zhang, D. G., Gui, J. & Su, R. Prediction of Cancer recurrence based on compact graphs of whole slide images. *Comput. Biol. Med.* **167**, 107663 (2023). 37931526 10.1016/j.compbiomed.2023.107663

[2] Tokuyama, N. et al. Prediction of non-muscle invasive bladder cancer recurrence using machine learning of quantitative nuclear features. *Mod. Pathol.* **35**, 533–538 (2022). 34716417 10.1038/s41379-021-00955-y PMC8964412

[3] Shalata, A. T. et al. Predicting recurrence of Non-Muscle-Invasive bladder cancer: current techniques and future trends. *Cancers* **14**(20), pp5019 (2022). 10.3390/cancers14205019 PMC9599984 36291803

[4] Barrios, W. et al. Bladder cancer prognosis using deep neural networks and histopathology images. *J. Pathol. Inf.* **13**, 100135 (2022). 10.1016/j.jpi.2022.100135 PMC9577122 36268091

[5] Johnson, K. B. et al. Precision medicine, AI, and the future of personalized health care. *Clin. Transl Sci.* **14**(1), 86–93 (2021). 32961010 10.1111/cts.12884 PMC7877825

[6] Leiserson, M. D. et al. Pan-cancer network analysis identifies combinations of rare somatic mutations across pathways and protein complexes. *Nat. Genet.* **47**, 106–114 (2015). 25501392 10.1038/ng.3168 PMC4444046

[7] Song, H. et al. Identification of Cancer driver genes by integrating mult omics data with graph neural networks. *Metabolites* **13**(3), 339. 10.3390/metabo13030339 (2023). 10.3390/metabo13030339 PMC10052551 36984779

[8] Peng, W., Tang, Q., Dai, W. & Chen, T. Improving cancer driver gene identification using multi-task learning on graph convolutional network. *Brief Bioinform.* **23**(1), bbab432 (2022). 10.1093/bib/bbab432 10.1093/bib/bbab432 34643232

[9] Das, S., Hayden, J., Sullivan, T. & Rieger-Christ, K. The roles of MiRNAs in predicting bladder Cancer recurrence and resistance to treatment. *Int. J. Mol. Sci.* **24**(2), 964 (2023). 36674480 10.3390/ijms24020964 PMC9864802

[10] Zhu, W. et al. Long noncoding RNAs in bladder cancer prognosis: A meta-analysis. *Pathol. Res. Pract.* **215**(6), 152429 (2019). 31064722 10.1016/j.prp.2019.04.021

[11] Smith, R. et al. Personalized Cancer prediction using gene expression profiling and deep learning techniques. *Cancer Res.* **80**(5), 1101–1110 (2020).

[12] Panchami. V. U. & Manish. T. I. Bladder cancer prediction using genetic algorithm and fuzzy rule-based system. *2021 International Conference on Communication, Control and Information Sciences (ICCISc)*, Idukki, India, 2021, pp. 1–6. 10.1109/ICCISc52257.2021.9484862

[13] Kırboğa, K. K. Bladder Cancer gene expression prediction with explainable algorithms. *Neural Comput. Application*. **36**, 1585–1597 (2024).

[14] Kim, S. Y. Discrete-time survival prediction using graph neural networks. *Bioengineering* **10**(9), 1046 (2023). 10.3390/bioengineering10091046 PMC10525217 37760148

[15] Chen, J., Pan, J., Zhu, Y. & Li, J. SLGNNCT: Synthetic lethality prediction based on knowledge graph for different cancers types. In: (eds Huang, D. S., Zhang, Q. & Guo, J.) Advanced Intelligent Computing in Bioinformatics. ICIC 2024. Lecture Notes in Computer Science, vol. 14881. Springer, Singapore, 10.1007/978-981-97-5689-6_14. (2024).

[16] Haitham, E. et al. A new approach for cancer prediction based on deep neural learning. *J. King Saud Univ. Comput. Inf. Sci.* **35**(6), 101565 (2023).

[17] Sun, Y. et al. Identification of 12 Cancer types through genome deep learning. *Sci. Rep.* **9**, 17256 (2019). 31754222 10.1038/s41598-019-53989-3 PMC6872744

[18] Tsai, I. J., Shen, W. C., Lee, C. L., Wang, H. D. & Lin, C. Y. Machine learning in prediction of bladder Cancer on clinical laboratory data. *Diagnostics (Basel)*. **12**, pp203 (2022). 10.3390/diagnostics12010203 PMC8774436 35054370

[19] Fu, Y., Sun, S., Shi, D. & Bi, J. Construction of endothelial cell signatures for predicting the diagnosis, prognosis, and immunotherapy response of bladder cancer via machine learning. *J. Cell. Mol. Med.* **28**(6), e18155. 10.1111/jcmm.18155 (2024). 38429911 10.1111/jcmm.18155 PMC10907833

[20] Ji, J. et al. Using machine learning to develop preoperative model for lymph node metastasis in patients with bladder urothelial carcinoma. *BMC Cancer*. **24**, 725. 10.1186/s12885-024-12467-4 (2024). 38872141 10.1186/s12885-024-12467-4 PMC11170799

[21] Li, Z., Wang, T. & Wang Machine-learning prediction of a novel diagnostic model using mitochondria-related genes for patients with bladder cancer. *Sci. Rep.* **14**, 9282. 10.1038/s41598-024-60068-9 (2024). 38654047 10.1038/s41598-024-60068-9 PMC11039685

[22] Li, C. et al. A machine learning computational framework develops a multiple programmed cell death index for improving clinical outcomes in bladder Cancer. *Biochem. Genet.*10.1007/s10528-024-10683-y (2024). 38353892 10.1007/s10528-024-10683-y

[23] Yan, R. et al. Histopathological bladder cancer gene mutation prediction with hierarchical deep multiple-instance learning. *Med. Image Anal.* **87**, PP102824 (2023). 10.1016/j.media.2023.102824 37126973

[24] Velmahos, C. S. et al. Using deep learning to identify bladder cancers with FGFR-Activating mutations from histology images. *Cancer Med.* **10**(14), 4805–4813. 10.1002/cam4.4044 (2021). 34114376 10.1002/cam4.4044 PMC8290253

[25] Po-Wei, S. & Por-Sen, C. Systems drug design for muscle invasive bladder cancer and advanced bladder cancer by genome-wide microarray data and deep learning method with drug design specifications. *Int. J. Mol. Sci.* **23**, 13869. 10.3390/ijms232213869. (2022). 10.3390/ijms232213869 PMC9692470 36430344

[26] Zhou, Y., Zheng, X., Sun, Z. & Wang, B. Analysis of bladder Cancer staging prediction using deep residual neural network, radiomics, and RNA-Seq from High-Definition CT images. *Genetic Res.*10.1155/2024/4285171 (2024). 10.1155/2024/4285171 PMC11074870 38715622

[27] Sun, Y. et al. Comprehensive genomic data preprocessing for GCNs in Cancer detection: coding and Non-Coding gene integration. *Sci. Rep.* **9**, 17256 (2019).31754222

[28] Han, Z. et al. Patient-Specific genomic interaction networks for Cancer driver gene prediction: integrating coding and Non-Coding genes. *Sci. Rep.* **7**, 4172 (2017).28646155

[29] Shanthamallu, U. S., Thiagarajan, J. J., Song, H. & Spanias, A. GrAMME: Semi supervised learning using multilayered graph attention models, in IEEE transactions on neural networks and learning systems. **31**(10), 3977–3988 (2020). 10.1109/TNNLS.2019.2948797 31725400

[30] Wang, J. et al. Deep graph neural network with attention mechanism to predict synergistic drug combinations. *Brief Bioinform.* **23**(1), bbab390 (2022). 10.1093/bib/bbab390 34571537

[31] Salehin, I. & Kang, D. K. A review on dropout regularization approaches for deep neural networks within the scholarly domain. *Electronics* **12**(14), 3106 (2023).

[32] Garbin, C., Zhu, X. & Marques, O. Dropout vs. batch normalization: An empirical study of their impact to deep learning. *Multimed. Tools Appl.* **79**, 12777–12815. 10.1007/s11042-019-08453-9 (2000).

[33] Li, X., Chen, S., Hu, X., Yang, J. & Recognition, P. Understanding the disharmony between dropout and batch normalization by variance shift. *2019 IEEE/CVF Conference on Computer Vision and (CVPR)* 2677–2685. 10.1109/CVPR.2019.00279 (2019).

[34] Peng, Y. et al. Novel GCN model using dense connection and attention mechanism for text classification. *Neural Process Lett.* **56**, 144 (2024).

[35] Weinstein, J. et al. The Cancer genome atlas Pan-Cancer analysis project. *Nat. Genet.* **45**, 1113–1120. 10.1038/ng.2764 (2013). 24071849 10.1038/ng.2764 PMC3919969

[36] Jassal, B. et al. The Reactome Pathway Knowledgebase. *Nucleic Acids Res.* **48**, D498–D503. 10.1093/nar/gkz1031 (2020). 10.1093/nar/gkz1031 PMC7145712 31691815

[37] Slenter, D. N. et al. Wiki pathways: a multifaceted pathway database bridging metabolomics to other omics research. *Nucleic Acids Res.* **46**(D1), D661–D667. 10.1093/nar/gkx1064 (2018). 29136241 10.1093/nar/gkx1064 PMC5753270

